# Keeping crispr in check: diverse mechanisms of phage-encoded anti-crisprs

**DOI:** 10.1093/femsle/fnz098

**Published:** 2019-05-11

**Authors:** Despoina Trasanidou, Ana Sousa Gerós, Prarthana Mohanraju, Anna Cornelia Nieuwenweg, Franklin L Nobrega, Raymond H J Staals

**Affiliations:** 1 Laboratory of Microbiology, Department of Agrotechnology and Food Sciences, Wageningen University and Research, Stippeneng 4, Wageningen 6708 WE, The Netherlands; 2 Kavli Institute of Nanoscience, Department of Bionanoscience, Delft University of Technology, Van der Maasweg 9, 2629 HZ Delft, The Netherlands

**Keywords:** crispr-cas, phage, genome editing, anti-crispr

## Abstract

CRISPR-Cas represents the only adaptive immune system of prokaryotes known to date. These immune systems are widespread among bacteria and archaea, and provide protection against invasion of mobile genetic elements, such as bacteriophages and plasmids. As a result of the arms-race between phages and their prokaryotic hosts, phages have evolved inhibitors known as anti-CRISPR (Acr) proteins to evade CRISPR immunity. In the recent years, several Acr proteins have been described in both temperate and virulent phages targeting diverse CRISPR-Cas systems. Here, we describe the strategies of Acr discovery and the multiple molecular mechanisms by which these proteins operate to inhibit CRISPR immunity. We discuss the biological relevance of Acr proteins and speculate on the implications of their activity for the development of improved CRISPR-based research and biotechnological tools.

## Teaser

In this review, we discuss our current understanding of phage-encoded inhibitors that block prokaryotic CRISPR-Cas systems, their mechanisms, structures and applications.

## INTRODUCTION

Viruses are ubiquitous entities co-existing with cellular life forms, present in almost all explored environments (Koonin and Dolja 2013). Viruses that infect bacteria (bacteriophages or phages) are the most abundant biological entities on the planet with population numbers in the order of 10^31^(Suttle 2005; Koonin and Dolja 2013; Guemes *et al*. 2016). The ability of phages to easily manoeuvre between different biomes, operating as vehicles of horizontal gene transfer (HGT), makes them major agents of evolution (Sano *et al*. 2004). Bacteriophages are classified based on their life-cycle into virulent and temperate. Virulent phages rely exclusively on productive infection cycles for propagation, which ultimately kills the host for the release of new viral particles that can engage in another round of infection. Temperate phages have the choice to multiply in their host cells leading to cell lysis or to integrate their phage genome into the bacterial chromosome as a prophage. Prophages are propagated passively by the replication machinery of the bacterial cell (Gandon 2016).

As a response to the constant threat of phage infection, a diverse arsenal of defence mechanisms has evolved in bacterial hosts. Because phages evolve rapidly to counter these immune systems (Drake *et al*. 1998), the hosts need to constantly evolve new means of self-protection, leading to a perennial arms-race between hosts and their phages (Forterre and Prangishvili 2009). The defence systems evolved by bacteria provide both innate and adaptive immunity against phage infection. Innate immunity systems interfere at different levels of the phage's infection cycle via receptor masking, superinfection exclusion (Sie), restriction–modification (R-M), bacteriophage exclusion (BREX), toxin–antitoxin (TA) modules, abortive infection (Abi), prokaryotic Argonautes (pAgos), production of anti-phage chemicals and defence island system associated with restriction–modification (DISARM) systems (Chopin, Chopin and Bidnenko 2005; Makarova *et al*. 2011; Makarova, Wolf and Koonin 2013; Samson *et al*. 2013; Goldfarb *et al*. 2015; Kronheim *et al*. 2018), whereas other remain to be characterized (Doron *et al*. 2018). Adaptive and heritable immunity is provided by Clustered Regularly Interspaced Short Palindromic Repeats (CRISPR)–CRISPR-associated (Cas) systems (Barrangou *et al*. 2007), which work as a fascinating complementation to the innate defence strategies.

Diverse variants of the CRISPR-Cas defence system are present in most of the sequenced genomes of archaea and half of those of bacteria (Makarova *et al*. 2013). A CRISPR-Cas locus typically consists of a CRISPR array and an operon of CRISPR-associated (*cas*) genes. The CRISPR array is composed of a series of short, partially palindromic and direct repetitive sequences (repeats) interspaced by variable sequences (spacers), originating from phage genomes or other invading mobile genetic elements (MGE), such as (conjugative) plasmids (Bolotin *et al*. 2005; Mojica *et al*. 2005; Pourcel, Salvignol and Vergnaud 2005; Shmakov *et al*. 2017). The *cas* genes encode for the Cas proteins, which are necessary for the generation of new spacers or are involved in the targeting of the MGE, as explained below. Collectively, these two elements of CRISPR-Cas systems mediate sequence-specific immunity against invasive MGEs (Brouns *et al*. 2008; Marraffini and Sontheimer 2008; Hale *et al*. 2009; Garneau *et al*. 2010).

The continuous arms-race between prokaryotic hosts and their cognate MGEs is speculated to be responsible for the rapid evolution of highly diverse CRISPR-Cas systems. The current CRISPR-Cas classification scheme distinguishes two broad classes based on the protein composition of the effector Cas complex. Class 1 systems (types I, III and IV) use multi-subunit Cas protein complexes for the recognition of targeted nucleic acids, while the less common class 2 systems (types II, V and VI) employ a single multi-domain effector protein complex that performs target recognition and cleavage. These classes are further subdivided into a total of six CRISPR types with 25 subtypes (Koonin, Makarova and Zhang 2017).

Despite substantial structural and functional diversity, all CRISPR-Cas systems mediate immunity through three distinct steps: adaptation, expression and interference (Mohanraju *et al*. 2016). During adaptation, short DNA fragments (known as protospacers) are acquired from invading MGEs and subsequently processed and inserted as spacers into the CRISPR locus, typically by the Cas1–Cas2 complex (Jackson *et al*. 2017). Next, during expression, the CRISPR array is transcribed as a long precursor CRISPR RNA (pre-crRNA) and the Cas proteins are expressed. The pre-crRNA is then processed within repeat regions to yield mature CRISPR RNAs (crRNAs) by dedicated Cas proteins and/or host factors (Brouns *et al*. 2008; Hale *et al*. 2008; Haurwitz *et al*. 2010; Deltcheva *et al*. 2011). The crRNAs are packaged with one or more Cas proteins into effector Cas complexes that scrutinise the microbial cell for potential invasion. Finally, during interference, the Cas complexes recognize complementary target sequences of invading MGEs by Watson–Crick base-pairing. Upon binding to a cognate target sequence, the complex either recruits a nuclease or stimulates its intrinsic nuclease activity to neutralize the invader (Brouns *et al*. 2008; Garneau *et al*. 2010; Westra *et al*. 2012). Type I, II and V CRISPR-Cas systems target DNA and rely on a short stretch (2 to 7 nucleotides) of conserved nucleotides adjacent to the protospacer, known as the protospacer-adjacent motif (PAM), for spacer selection during adaptation and target identification during interference (Mojica *et al*. 2005; Marraffini and Sontheimer 2008). The PAM allows for self/nonself discrimination, as its absence in the CRISPR array prevents autoimmunity and self-cleavage.

In response to the microbial antiviral defence mechanisms, phages have evolved numerous mechanisms to overcome prokaryotic immunity. Phage evasion from prokaryotic CRISPR-Cas systems was first found to rely on mutational drifts, predominantly occurring in regions that require perfect complementarity between the crRNA and the protospacer (so-called seed region) for interference, or in the PAM sequences (Deveau *et al*. 2008; Sun *et al*. 2013; Bondy-Denomy *et al*. 2015). Depending on the location of the mismatch (between the crRNA and the protospacer), a single mutation can be sufficient to abolish CRISPR-Cas immunity (Deveau *et al*. 2008; Semenova *et al*. 2011). Deletion of the protospacer sequence and/or the PAM have also been shown to provide an effective way for phages to escape CRISPR-Cas targeting, despite the potential of imposing a fitness cost (Deveau *et al*. 2008). Similar to the evasion strategy from R-M systems, phages can also modify their bases with hydroxymethylcytosine (HMC) and its bulkier glycosylated form to reduce target binding affinity and thereby protect from CRISPR-mediated targeting by both type I and type II systems (Bryson *et al*. 2015; Vlot *et al*. 2018), whereas other modifications do not disturb Cas9 recognition (Yaung, Esvelt and Church 2014). Finally, some phages encode their own CRISPR locus that targets host antiviral genomic regions, such as chromosomal (defence) islands (Seed *et al*. 2013).

The first examples of phage-encoded anti-CRISPR (Acr) proteins were found in class 1 type I-F and I-E systems of *Pseudomonas aeruginosa* (Bondy-Denomy *et al*. 2013; Pawluk *et al*. 2014). Acr proteins have distinct sequences (Tables 1 and 2), structures (Maxwell *et al*. 2016; Wang *et al*. 2016a; Harrington *et al*. 2017) and mechanisms (Bondy-Denomy *et al*. 2015) and they provide phages with a direct and specific means to inhibit targeting by the CRISPR-Cas system. To date, 45 unique families of Acr proteins have been discovered, and categorized into class 1 (Table 1) and class 2 (Table 2) CRISPR-Cas inhibitors. These highly diverse and small (typically 50–330 amino acids) proteins do not share much sequence or protein domain similarity to each other or to any protein of known function (Marino *et al*. 2018).

**Figure 1.**
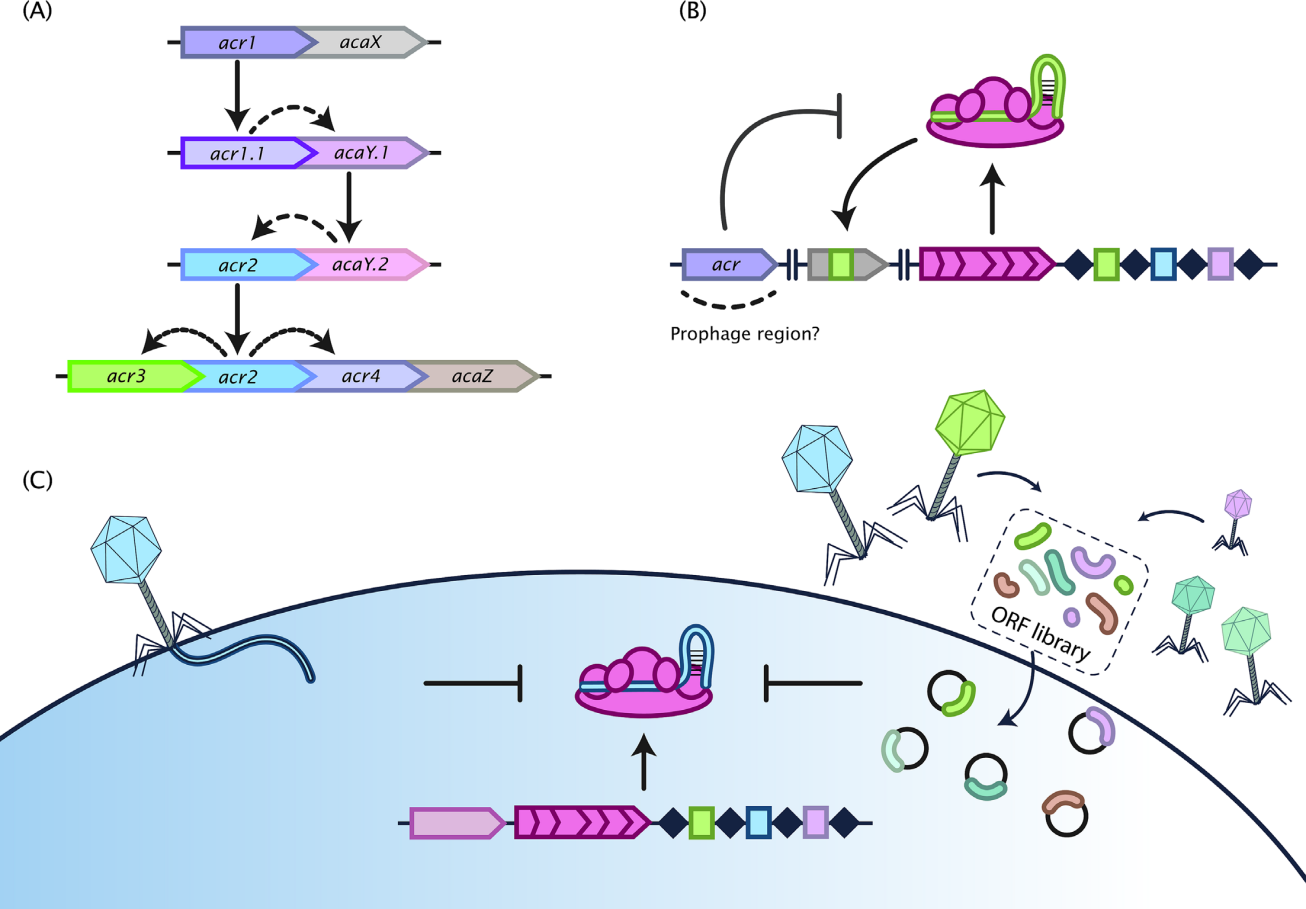
Different discovery and testing methods of Acrs. **(A)** Guilt-by-association discovery method (Pawluk *et al*. 2016a). This discovery method is based on the strong co-occurrence and clustering of *acr* and *aca* genes through proximity and homology searches. In this example, homology searches using the *acr1* gene yields its homologue *acr1.1*. Inspection of genes in close proximity yielded *acaY.1*, which in turn can be used for further iterative rounds of *acr* and/or *aca* gene discovery. Both *acr* and *aca* genes typically appear in clusters leading to the discovery of new *acr* and *aca* genes. **(B)** The self-targeting discovery method (Rauch *et al*. 2017). The presence of a self-targeting spacer (in green) within the CRISPR array hints at the presence of a (set of) *acr* gene(s) (in purple) somewhere within the host's genome, often within prophage regions. **(C)** Low- and high-throughput functional assays to identify phage-encoded Acrs. In a low-throughput assay, individual phages are used to screen for anti-CRISPR activity in hosts with a CRISPR-Cas system targeting the phage (left) using (for example) plaque assays. High-throughput screening can be performed by transforming phage ORF libraries that are placed on a plasmid containing a protospacer. Successful transformants can be screened further to pinpoint the gene with the Acr activity within the collection of ORFs.

**Table 1.** Class 1 anti-CRISPR protein families.

Family	Size (aa)	Origin (characterized homolog)	Method of discovery	Accession number	CRISPR-Cas type inhibited [organism]	Mode-of-action	Structure	PDB code	References
AcrIC1	190	*Moraxella bovoculi* prophage	Self-targeting, Guilt-by-association [*acrIF11*]	AKG19229.1	I-C [Mbo]	—	—	—	(Marino *et al*. 2018)
AcrID1	98	*Sulfolobus islandicus* rudivirus 3	Functional assays	YP_009272954.1	I-D [Sis]	Binds as a dimer to the Cas10d, mimicking DNA (blocks DNA binding)	Compact dimeric αβ-sandwich; each monomer 5-stranded antiparallel β-sheet + 2 α-helices at one side of the β-sheet	6EXP	(He *et al*. 2018)
AcrIE1	100	*Pseudomonas aeruginosa* phage JBD5	Functional assays	YP_007392738.1	I-E [Pae]	Binds as a dimer to the Cas3 (blocks DNA cleavage)	Elongated dimeric structure; each monomer 1 antiparallel β-sheet + 3 α-helices	6ARZ, 6AS4	(Pawluk *et al*. 2014), (Pawluk *et al*. 2017)
AcrIE2	84	*P. aeruginosa* phage JBD88a	Functional assays	YP_007392439.1	I-E [Pae]	—	—	—	(Pawluk *et al*. 2014)
AcrIE3	68	*P. aeruginosa* phage DMS3	Functional assays	YP_950454.1	I-E [Pae]	Probably binds to the Cascade (blocks DNA binding)	—	—	(Pawluk *et al*. 2014)
AcrIE4	52	*P. aeruginosa* phage D3112	Functional assays	NP_938238.1	I-E [Pae]	—	—	—	(Pawluk *et al*. 2014)
AcrIE5	65	*Pseudomonas otitidis* mobile genetic element	Guilt-by-association [*aca1*]	WP_074973300.1	I-E [Pae]	—	—	—	(Marino *et al*. 2018)
AcrIE6	79	*P. aeruginosa* mobile genetic element	Guilt-by-association [*aca1*]	WP_087937214.1	I-E [Pae]	—	—	—	(Marino *et al*. 2018)
AcrIE7	106	*P. aeruginosa* mobile genetic element	Guilt-by-association [*aca1*]	WP_087937215.1	I-E [Pae]	—	—	—	(Marino *et al*., 2018)
AcrIF1	78	*P. aeruginosa* phage JBD30	Functional assays	YP_007392342.1	I-F [Pae, Pec]	2–3 copies interact with the hexameric Cas7f spine of the Cascade (block DNA binding)	4-stranded antiparallel β-sheet + 2 α-helices at one side of the β-sheet	2LW5, 5UZ9, 6ANV, 6B46	(Bondy-Denomy *et al*. 2013), (Guo *et al*., 2017), (Chowdhury *et al*. 2017)
AcrIF2	90	*P. aeruginosa* phage D3112	Functional assays	NP_938237	I-F [Pae, Pec]	Binds to the Cas5f:Cas8f tail of the Cascade, mimicking DNA (blocks DNA binding)	4-stranded antiparallel β-sheet + 2 antiparallel α-helices at either side of the β-sheet	5UZ9, 6B47	(Bondy-Denomy *et al*. 2013), (Guo *et al*., 2017), (Chowdhury *et al*. 2017)
AcrIF3	139	*P. aeruginosa* phage JBD88a	Functional assays	YP_007392440.1	I-F [Pae]	Binds as a dimer to the Cas3, preventing its recruitment to the Cascade-dsDNA (blocks DNA binding) or spacer acquisition by the Cas1–2/3 complex (blocks adaptation)	Dimeric structure; each monomer 6 α-helices	5GNF, 5GQH, 5B7I	(Bondy-Denomy *et al*. 2013), (Vorontsova *et al*. 2015), (Wang *et al*. 2016a), (Wang *et al*. 2016b)
AcrIF4	100	*P. aeruginosa* phage JBD26	Functional assays	WP_016068584.1	I-F [Pae]	Binds to the Cascade (blocks DNA binding)	—	—	(Bondy-Denomy *et al*. 2013)
AcrIF5	79	*P. aeruginosa* phage JBD5	Functional assays	YP_007392740.1	I-F [Pae]	—	—	—	(Bondy-Denomy *et al*. 2013)
AcrIF6	100	*P. aeruginosa* prophage	Guilt-by-association [*aca1*]	WP_043884810	I-F [Pae, Pec], I-E [Pae]	—	—	—	(Pawluk *et al*.2016a)
AcrIF7	83	*P. aeruginosa* prophage	Guilt-by-association [*aca1*]	ACD38920.1	I-F [Pae, Pec]	—	—	—	(Pawluk *et al*. 2016a)
AcrIF8	92	*Pectobacterium carotovorum* phage ZF40	Guilt-by-association [*aca2*]	AFC22483.1	I-F [Pae, Pec]	—	—	—	(Pawluk *et al*. 2016a)
AcrIF9	68	*Vibrio parahaemolyticus* mobile genetic element	Guilt-by-association [*aca2*]	WP_031500045.1	I-F [Pae, Pec]	—	—	—	(Pawluk *et al*. 2016a)
AcrIF10	97	*Shewanella xiamenensis* prophage	Guilt-by-association [*aca2*]	KEK29119	I-F [Pae, Pec]	Binds to the Cas7f:Cas8f tail, mimicking DNA (blocks DNA binding)	4-stranded antiparallel β-sheet + 3 antiparallel α-helices at one side of the β-sheet	6ANW, 6B48	(Pawluk *et al*. 2016a), (Guo *et al*. 2017)
AcrIF11	132	*P. aeruginosa* mobile genetic element	Guilt-by-association [*aca1*]	WP_038819808.1	I-F [Pae]	—	—	—	(Marino *et al*. 2018)
AcrIF12	124	*P. aeruginosa*	Guilt-by-association [*aca4*]	ABR13388.1	I-F [Pae]	—	—	—	(Marino *et al*. 2018)
AcrIF13	115	*Moraxella catarrhalis* prophage	Self-targeting, Guilt-by-association [*acrIF11*]	EGE18854.1	I-F [Mbo]	—	—	—	(Marino *et al*. 2018)
AcrIF14	124	*M. catarrhalis* phage Mcat5	Self-targeting, Guilt-by-association [*acrIF11*]	AKI27193.1	I-F [Mbo]	—	—	—	(Marino *et al*. 2018)
AcrIE4-F7	119	*Pseudomonas citronellolis* mobile genetic element	Guilt-by-association [*aca1*]	WP_064584002.1	I-E [Pae], I-F [Pae]	—	—	—	(Marino *et al*. 2018)

**Table 2.** Class 2 anti-CRISPR protein families.

Family	Size (aa)	Origin (characterized homolog)	Method of discovery	Accession number	CRISPR-Cas type inhibited [organism]	Mode-of-action	Structure	PDB code	References
AcrIIA1	149	*Listeria monocytogenes* prophage J0161a	Self-targeting	WP_003722518.1	II-A [Lmo]	Recognizes nucleic acids (putative transcriptional regulation)	Dimeric structure with pseudo 2-fold symmetry; each monomer 5 α-helices + 13_10_ helix at N-terminus and 3 α-helices + 23_10_ helices at C-terminus (all helical 2-domain)	5Y6A	(Rauch *et al*. 2017), (Ka *et al*. 2018)
AcrIIA2	123	*L. monocytogenes* prophage J0161a	Self-targeting	WP_003722517.1	II-A [Lmo, Spy]	Binds to the PAM-interacting, the WED, the HNH, and the REC2 domains (blocks DNA recognition, binding/unwinding, and cleavage, respectively)	Bent 4-stranded antiparallel β-sheet + 2 α-helices at either side of the β-sheet	6MCB, 6MCC, 6IFO	(Rauch *et al*. 2017), (Jiang *et al*. 2019), (Liu *et al*. 2018)
AcrIIA3	125	*L. monocytogenes* prophage SLCC2482	Self-targeting, Guilt-by-association [*acrIIA1*]	WP_014930691.1	II-A [Lmo]	—	—	—	(Rauch *et al*. 2017)
AcrIIA4	87	*L. monocytogenes* prophage J0161b	Self-targeting, Guilt-by-association [*acrIIA1*]	WP_003723290.1	II-A [Lmo, Spy]	Binds to the PAM-interacting, the Topo-homology, and the RuvC domains (blocks DNA recognition, binding/unwinding, and cleavage, respectively)	3-stranded antiparallel β-sheet + 3 α-helices at one side of the β-sheet + 13_10_ helix	5XN4, 5XBL, 5VW1, 5VZL	(Rauch *et al*. 2017), (Kim *et al*. 2018), (Dong *et al*. 2017), (Yang and Patel, 2017), (Shin *et al*. 2017)
AcrIIA5	140	*Streptococcus thermophilus* (virulent) phage D4276	Functional assays	ASD50988.1	II-A [Sth1, Sth3, Spy]	—	—	—	(Hynes *et al*. 2018)
AcrIIA6	183	*S. thermophilus* (virulent) phage D1811	Functional assays, Guilt-by-association [*acrIIA5*]	MH000604	II-A [Sth1]	—	Dimeric structure; each monomer 4-stranded antiparallel β-sheet + 8 α-helices	6EYX,	
6EYY									(Hynes *et al*. 2018)
AcrIIA7	103	Human gut metagenomic libraries	Synthetic genetic circuit for screening of metagenomic libraries	LR030272	II-A [Spy]	—	—	—	(Uribe *et al*. 2019)
AcrIIA8	105	Human gut metagenomic libraries	Synthetic genetic circuit for screening of metagenomic libraries	LR030270	II-A [Spy]	—	—	—	(Uribe *et al*. 2019)
AcrIIA9	141	Human gut metagenomic libraries	Synthetic genetic circuit for screening of metagenomic libraries	LR030269	II-A [Spy]	—	—	—	(Uribe *et al*. 2019)
AcrIIA10	109	Soil metagenomic libraries	Synthetic genetic circuit for screening of metagenomic libraries	LR030271	II-A [Spy]	—	—	—	(Uribe *et al*., 2019)
AcrIIC1	86	*Neisseria meningitidis* mobile genetic element	Guilt-by-association [*aca2*]	WP_049360089.1	II-C [Nme,Cje, Geo, Hpa, Smu]	Binds to the HNH active site (allows DNA binding, blocks DNA cleavage)	5-stranded β-bundle interspaced by 2 α-helices	5VGB	(Pawluk *et al*. 2016b), (Harrington *et al*. 2017), (Lee *et al*. 2018), (Zhu *et al*. 2019)
AcrIIC2	123	*N. meningitidis* prophage	Guilt-by-association [*aca3*]	WP_042743678.1	II-C [Nme, Hpa, Smu, SauCas9, SpyCas9, FnoCas9, CjeCas9]	Binds to the bridge helix (BH)-REC1 region (blocks DNA binding)	Dimeric structure; each monomer 6-stranded antiparallel β-sheet (half-barrel structure) flanked by 2 α-helices (the C-terminal α-helix is embedded into the half-barrel)	6J9K, 6J9L, 6J9M	(Pawluk *et al*. 2016b), (Lee *et al*. 2018), (Zhu *et al*. 2019)
AcrIIC3	116	*N. meningitidis* prophage	Guilt-by-association [*aca3*]	WP_042743676.1	II-C [Nme, Hpa, Smu]	Binds to the HNH domain opposite to the active site, and the REC lobe (hinders DNA binding, blocks DNA cleavage, forces Cas9 dimerization)	4-stranded antiparallel β-sheet + 3 α-helices at either side of the β-sheet	6J9N	(Pawluk *et al*. 2016b), (Lee *et al*. 2018), (Zhu *et al*. 2019)
AcrIIC4	88	*Haemophilus parainfluenzae* prophage	Guilt-by-association [*aca* not reported]	WP_049372635	II-C [Nme, Hpa, Smu]	Binds to the Cas9 (blocks DNA binding)	—	—	(Lee *et al*. 2018)
AcrIIC5	130	*Simonsiella muelleri* transfer element	Guilt-by-association [*aca* not reported]	WP_002642161.1	II-C [Nme, Hpa, Smu]	Binds to the Cas9 (blocks DNA binding)	—	—	(Lee *et al*. 2018)
AcrVA1	170	*Moraxella bovoculi* prophage	Self-targeting, Guilt-by-association [*acrIF11*]	WP_046701302.1	V-A [Mbo, Asp, Lba, Fno]	—	—	—	(Marino *et al*. 2018), (Watters *et al*. 2018)
AcrVA2	322	*M. bovoculi* prophage	Self-targeting, Guilt-by-association [*acrIF11*]	AKG19228.1	V-A [Mbo]	—	—	—	(Marino *et al*. 2018)
AcrVA3	168	*M. bovoculi* prophage	Self-targeting, Guilt-by-association [*acrIF11*]	AKG19230.1	V-A [Mbo], I-C [Mbo]	—	—	—	(Marino *et al*. 2018)
AcrVA4	234	*M. bovoculi* prophage	Self-targeting	WP_046699156.1	V-A [Mbo, Lba]	—	—	—	(Watters *et al*. 2018)
AcrVA5	92	*M. bovoculi* prophage	Self-targeting	WP_046699157.1	V-A [Mbo, Lba]	—	—	—	(Watters *et al*. 2018)
Csx27	201	*Bergeyella zoohelcum*	Guilt-by-association [*cas13b*]	WP_034985946.1	VI-B	—	—	—	(Smargon *et al*. 2017)

Here, we explore the biological relevance and detail the recent insights into the molecular mechanisms and structures of anti-CRISPR proteins. We also address the development of anti-CRISPRs as ‘off-switches’ for genome editing and discuss the impact of their use in other biotechnological applications.

## BIOLOGICAL RELEVANCE OF ANTI-CRISPR PROTEINS

The emergence of widespread, specialized and highly diverse phage-encoded proteins that thwart CRISPR-Cas immunity, suggests that Acr proteins play an important role in phage biology. The first identified Acr proteins were shown to inactivate the type I-F CRISPR-Cas system of *P. aeruginosa*, halting the host CRISPR machinery upon phage infection (Bondy-Denomy *et al*. 2013). However, finding other Acr proteins by homology searches proved to be a challenging task due to their low sequence similarity. Instead, it was noted that the genomic neighbourhood of *acr* genes had interesting similarities that could be exploited to discover new Acrs. Typically, many *acr* genes co-occur with a group of genes that were collectively called ‘anti-CRISPR-associated genes’ (*aca*’s) (Pawluk *et al*. 2016a). To date, seven *aca* genes have been identified (Table 3). While the function of *aca*’s is not yet understood, these genes often encode for a protein containing a helix-turn-helix (HTH) motif, suggesting they fulfil a regulatory function (Pawluk *et al*. 2016a). Nevertheless, the presence of *aca*’s has been instrumental in finding new Acr proteins and vice-versa, a method that is now known as ‘guilt-by-association’ (Fig. 1A). In addition, the occurrence of a so-called ‘self-targeting’ spacer (i.e. a spacer that targets the host's own genome) within the CRISPR array is often indicative of a suppressed CRISPR-Cas system due to the presence of a (prophage encoded) *acr* gene (Rauch *et al*. 2017) (Fig. 1B). Furthermore, novel Acr proteins can be found using (high-throughput) screening and testing assays, including transformation of metagenomic libraries in an Acr-selection strain (Fig. 1C) either combined or not with synthetic genetic circuit-based selection for CRISPR-Cas suppression activity (Uribe *et al*. 2019).

**Table 3.** Anti-CRISPR-associated (*aca*) genes used in the guilt-by-association approach.

Name	Size (aa)	Accession number	References
*aca1*	79	YP_007392343	(Bondy-Denomy *et al*. 2013)
*aca2*	125	WP_019933869.1	(Pawluk *et al*. 2016a)
*aca3*	70	WP_049360086.1	(Pawluk *et al*. 2016a)
*aca4*	67	ABR13385.1	(Marino *et al*. 2018)
*aca5*	60	WP_039494319.1	(Marino *et al*. 2018)
*aca6*	65	WP_035450933.1	(Marino *et al*. 2018)
*aca7*	68	WP_064702654.1	(Marino *et al*. 2018)

The high diversity of the Acr proteins, their ability to inhibit different (sub)types of CRISPR-Cas systems (I-C, I-D, I-E, I-F, II-A, II-C, V-A, VI-B) (Smargon *et al*. 2017; He *et al*. 2018; Marino *et al*. 2018), their widespread presence and their usual coexistence in the same locus, demonstrate the strong evolutionary pressure that CRISPR-Cas systems exert in Acr arsenal diversification, and vice versa, meeting the Red Queen Hypothesis on the continuous shaping of the host-invader dynamics (Westra *et al*. 2015; van Houte *et al*. 2016).

The origin of Acr proteins remains to be understood, but it is hypothesized that these proteins do not share a common ancestor due to their low structural similarity (Pawluk *et al*. 2016a). While it is theorized they represent a product of *de novo* evolution from intergenic regions (Tautz, 2014; Stanley and Maxwell 2018), parallel studies show that they might have derived from other bacterial or viral proteins, as specific nuclease inhibitors, regulatory or even phage capsid proteins (Stanley and Maxwell 2018; Stone *et al*. 2018). Due to their function, *acr* genes were classified as accessory, or ‘morons’, since they are not strictly necessary in a phage lifecycle (Juhala *et al*. 2000; Brussow, Canchaya and Hardt 2004; Borges, Davidson and Bondy-Denomy 2017). However, when facing specific CRISPR-active hosts, the presence of these genes was shown to increase the fitness of Acr-positive phage populations (Bondy-Denomy *et al*. 2013; Pawluk *et al*. 2016a). While previous studies show the activity of CRISPR-Cas systems *in vivo* can clear a targeted phage in as little as 2 min (Garneau *et al*. 2010; Borges *et al*. 2018), the presence of Acr proteins seems to decrease or completely abolish bacterial immunity, classifying it as a major CRISPR-counteracting mechanism for successful phage infection and replication. However, the fast-acting nature of CRISPR-Cas limits the potential of a single phage to overcome the host's defence by Acr activity. Recently, it was shown that even though CRISPR-Cas systems are partially affected by the expression of Acr proteins, the latter are not able to confer full protection to their phage associated genome upon a single infection (Borges *et al*. 2018; Landsberger *et al*. 2018) Instead, a critical Acr concentration inside each single cell is necessary for successful host immunosuppression, allowing posterior lytic re-infection or genomic integration of temperate phages (Borges *et al*. 2018; Landsberger *et al*. 2018). It was then demonstrated that a single clonal phage population could inactivate CRISPR-Cas immunity through phage cooperation, where failed infections from ‘sacrificial Acr donors’ allow accumulation of Acr inhibitors inside a cell, which, upon a certain threshold, leads the ‘acceptor’ phages to successfully infect and amplify (Borges *et al*. 2018; Landsberger *et al*. 2018). The quantitative demand of Acr proteins for full host immunosuppression postulated that phage concentration has a key role on CRISPR evasion, which is inversely proportional to the strength of each Acr protein (Borges *et al*. 2018; Landsberger *et al*. 2018).

The existence of Acr proteins might also explain the incomplete, absent or deficient CRISPR-Cas systems found in bacteria (Stanley and Maxwell 2018). Prophage integration into the host chromosome and consistent Acr expression might result in CRISPR-Cas inactivating mutations, loss of *cas* genes and even complete loss of CRISPR-Cas systems (Stanley and Maxwell 2018). Interestingly, although it is evident that Acr proteins are relevant for the phage it originated from, bacteria might also benefit from the stable expression of these proteins (e.g. from prophage regions). For example, inhibition of CRISPR-Cas immunity might enhance HGT in these hosts, which can have a positive contribution to bacterial fitness upon acquisition of beneficial foreign genetic material (Bondy-Denomy *et al*. 2013; Jiang *et al*. 2013; Pawluk *et al*. 2014; Borges *et al*. 2018; Stanley and Maxwell 2018).

## MECHANISMS AND STRUCTURES OF ANTI-CRISPR PROTEINS

**Figure2.**
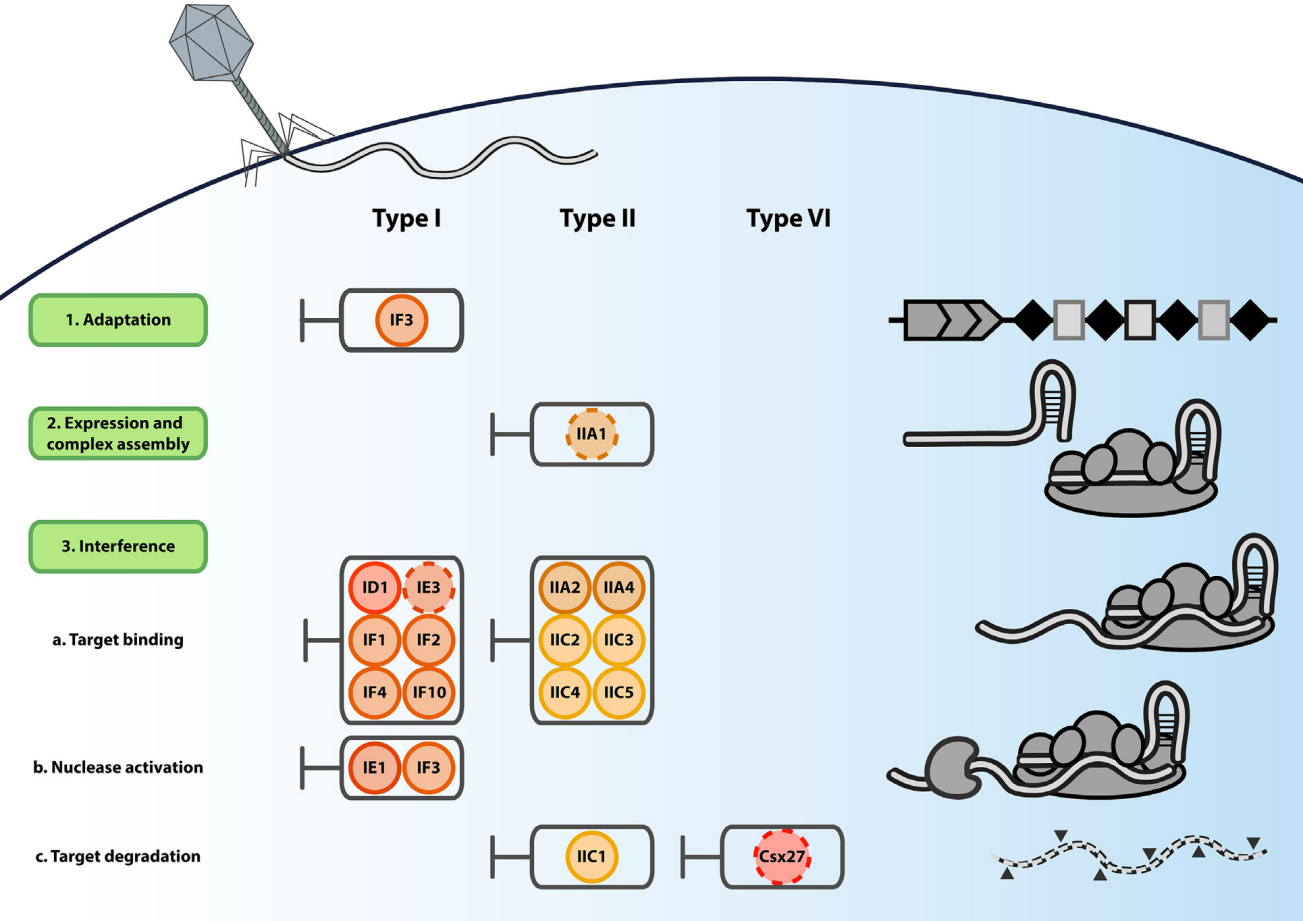
Schematic overview of the different Acrs and their mechanisms. The green boxes on the left show the different stages of CRISPR-Cas immunity. The columns indicate which CRISPR-Cas type is suppressed by which (group of) Acrs. Acrs are depicted as circles with their abbreviated names (e.g. AcrIF3 is abbreviated to IF3). A dashed line indicates a suggested role for the particular Acr or that the Acr mechanism remains to be elucidated. Note that most Acrs appear to suppress the interference stage, whereas only one Acr (AcrIF3) suppressed different stages.

Over the last six years, a series of studies interlacing genetic, biochemical and structural analyses have elucidated the mechanism of action of 12 Acr proteins from different families. Although many Acrs remain to be tested for anti-adaptation activity, the vast majority of the currently characterized Acr proteins act at the interference phase by directly blocking target DNA binding or cleavage (Fig. 2). These two general modes-of-action are spread among both class 1 and class 2 Acr proteins, with distinct molecular mechanisms (Borges, Davidson and Bondy-Denomy 2017).

### Class 1 Anti-CRISPR Proteins

The class 1 Acr proteins studied up till now all impede type I (subtype C, D, E or F) CRISPR-Cas systems (Table 1). Two mechanistic routes have been described for Acrs to perturb CRISPR interference: the most common is the direct interaction with the Cascade surveillance complex to prevent DNA binding (Bondy-Denomy *et al*. 2015; van Erp *et al*. 2015; Maxwell *et al*. 2016; Chowdhury *et al*. 2017; Guo *et al*. 2017; Peng *et al*. 2017), while the less common involves the direct interaction with the effector nuclease Cas3, which typically gets recruited upon successful target binding by the Cascade, to block DNA cleavage (Pawluk *et al*. 2014; Bondy-Denomy *et al*. 2015; Pawluk *et al*. 2016a; Wang *et al*. 2016a; Wang *et al*. 2016b; Pawluk *et al*. 2017).

### A) Preventing DNA Binding via Interaction with the Cascade Complex

#### Steric occlusion of DNA binding

AcrIF1 from *P. aeruginosa* phage JBD30 binds along the hexameric Cas7f spine with a stoichiometry of 2–3 molecules per Cascade complex (Bondy-Denomy *et al*. 2015). Several high-resolution cryo–electron microscopy and nuclear magnetic resonance (NMR) studies combined with site-directed mutagenesis indicated that AcrIF1 molecules bind tightly at different positions of the *P. aeruginosa* Cascade complex. More specifically, two AcrIF1 monomers sit on the Cas7f.4 and Cas7f.6 thumbs (Tyr6, Tyr20 and Glu31 lying on a single interaction surface of each monomer interact with the conserved Lsy85 on the Cas7f thumb), resulting in a conformational change that sterically blocks access of the crRNA guide to the target DNA, while a possible third monomer binds to a Cas7 region in close proximity to the Cas8f-Cas5f tail, which is crucial for target DNA binding (Bondy-Denomy *et al*. 2015; Maxwell *et al*. 2016; Chowdhury *et al*. 2017; Guo *et al*. 2017; Peng *et al*. 2017; Gabrieli *et al*. 2018).

#### DNA mimicry

While AcrIF1 exploits three different binding modes to disrupt target DNA recognition by the Cascade complex, AcrIF2 from *P. aeruginosa* phage D3112 mediates inhibition by interacting with a single site within the complex. AcrIF2 directly competes with the target DNA for a positively charged binding interface on the Cas5f:Cas8f tail between the Cas7f.6 thumb and the Cas8f hook, a region called the ‘lysine-rich vise’ (Bondy-Denomy *et al*. 2015). The small acidic AcrIF2 protein behaves as a DNA mimic, as the numerous acidic residues on its surface adopt a pseudo-helical distribution, resembling a double-stranded DNA (dsDNA) molecule. The interaction sites of AcrIF2 and DNA on the Cas5f:Cas8f heterodimer overlap partially, and AcrIF2 binding shoves the Cas8f hook away from the DNA-association pocket, sterically hampering the access of the dsDNA to the Cascade complex. Additional interactions of AcrIF2 with basic residues crucial for DNA binding further ensure obstruction of target DNA binding (van Erp *et al*. 2015; Chowdhury *et al*. 2017; Guo *et al. et al*. 2017; Peng *et al*. 2017). Given the close proximity of the interaction sites of AcrIF2 and the third AcrIF1 monomer, when cooperating, AcrIF1 exhibits a maximum of two binding modes while AcrIF2 activity remains intact (Peng *et al*. 2017).

Similar to AcrIF2, AcrIF10 from *Shewanella xiamenensis* prophage also mimics DNA by occupying a region on the Cas5f:Cas8f heterodimer that closely overlaps with the binding site of AcrIF2 (possibly Cas8f K71 and R78, Cas5f R90 and Cas7f K299). However, instead of wrenching the Cas8f hook away, AcrIF10 triggers a partially closed state of the hook swinging it toward Cas7.6f (similar but smaller than the movement caused by DNA binding), displaying the conformational flexibility of this domain and implying the need of additional interactions for absolute closure of the hook. Intriguingly, AcrIF10 and dsDNA display different charge profiles on the interaction surface; nevertheless, they interact with closely overlapping regions in the Cascade complex to prevent DNA binding (Guo *et al*. 2017).

The first archaeal Acr protein identified, AcrID1, was shown to directly interact as a homodimer with two copies of the large subunit (Cas10d) of the type I-D Cascade complex in *Sulfolobus islandicus*. The strong negatively charged surface of this protein suggests that it may behave as a DNA mimic, such as AcrIF2. In addition, conserved residues on the surface of AcrID1, such as Glu21, Lys34, Tyr55, Glu81, Arg92 and Trp91, may have a key role in inter-protein interactions. However, the exact mechanism of AcrID1 still remains to be explored (He *et al*. 2018).

#### Unknown mechanisms

AcrIE3 and AcrIF4 from *P. aeruginosa* phage DMS3 and JBD26, respectively, have been shown to associate with the Cascade complex to hinder DNA binding, though via an unknown mechanism (Pawluk *et al*. 2014; Bondy-Denomy *et al*. 2015).

### B) Preventing DNA Cleavage via Interaction with the Cas3 Nuclease

#### Disruption of binding to the Cascade:dsDNA chimera

Cryo-electron microscopy and X-ray crystallography demonstrated that the AcrIF3 protein from *P. aeruginosa* phage JBD5 forms a homodimer that binds to the Cas3 nuclease (Wang *et al*. 2016a; Wang *et al*. 2016b). High binding affinity was observed, since more than half of the AcrIF3 surface interacts with the Cas3 protein, forming several hydrogen bonds and hydrophobic interactions. As a consequence, the interaction sites for both the non-complementary DNA strand and the Cascade complex are blocked. Specifically, one AcrIF3 monomer occupies the helicase domain (HD) and the Linker region of Cas3, while the other monomer relates to the C-terminal domain (CTD) (Tyr97, Trp93, and a large network of hydrogen bonds), which altogether constitute the internal cleft of the Cas3 structure. By covering this cleft, AcrIF3 disrupts association with the target DNA (non-complementary strand) and locks the ATP-dependent Cas3 nuclease in an inactive ADP-bound form (Wang *et al*. 2016a; Wang *et al*. 2016b). Noteworthy, the Cas3 effector nuclease/helicase is fused to the Cas2 protein in type I-F systems and thereby forms an integral part of the type I-F (primed) adaptation machinery, hinting at the functional link between adaptation and interference, as shown recently (Kunne *et al*. 2016; Staals *et al*. 2016; Fagerlund *et al*. 2017). Interestingly, AcrIF3 dimer binds to the opposite site of Cas2, thus not influencing the assembly of the Cas1-Cas2-Cas3 complex (Rollins *et al*. 2017). However, the dimer obstructs the recruitment of the Cascade:dsDNA chimera to Cas3, preventing the generation of precursor protospacer DNA. Consequently, AcrIF3 blocks both primed spacer acquisition and crRNA interference (Vorontsova *et al*. 2015; Wang *et al*. 2016b).

#### Unknown mechanisms

Akin to AcrIF3, AcrIE1 from *P. aeruginosa* phage JBD5 directly associates with the ATP-dependent Cas3 nuclease, without affecting the binding ability of the Cascade to the target DNA. Due to the structural homology between Cas3 proteins of type I-F and I-E CRISPR-Cas systems, it is likely that AcrIF3 and AcrIE1 either adopt distinct modes of binding to the same surface or target unrelated regions on the Cas3 protein, hindering target DNA cleavage (Pawluk *et al*. 2014; Pawluk *et al*. 2017).

### Class 2 anti-CRISPR proteins

Class 2 Acr proteins have been discovered for type II (subtype A and C), type V (subtype A) and type VI (subtype B) CRISPR-Cas systems (Table   2). Almost all type II Acrs characterized to date directly interact with the Cas9 endonuclease, although by distinct mechanisms, as described below.

### A) Preventing DNA Binding via Interaction with the Cas9 Protein

#### DNA mimicry and steric occlusion of DNA binding and cleavage

Both AcrIIA2 and AcrIIA4 from *Listeria monocytogenes* prophage J0161a/b have been demonstrated *in vivo* and *in vitro* to directly interact with single-guide RNA (sgRNA)-loaded SpyCas9, abolishing DNA binding and cleavage (Dong *et al*. 2017; Rauch *et al*. 2017; Yang and Patel 2017; Basgall *et al*. 2018). Biochemical and structural studies have indicated that AcrIIA4 binds to several regions within SpyCas9. First, AcrIIA4 (Asp14, Asp37, Glu40, Asp69 and Glu70) associates with the PAM-interacting domain (Glu1108, Ser1109, Ser1216, Lys1200, Arg1335 and Arg1333) through an acidic surface that mimics a negatively-charged dsDNA molecule, thereby hampering the initial PAM searching stage. Second, AcrIIA4 (Asp14 and Asn36) interacts with the Topo-homology domain (Glu1108, Ser1109 and Ser1136), also known as DNA-melting region, putatively preventing DNA binding or unwinding. Third, AcrIIA4 (Leu19–Gln29) forms numerous surface complementarities with the concave surface of SpyCas9 at the RuvC domain (Asn767, Thr13, Ala764 and Arg976), abrogating the endonuclease activity. In addition, AcrIIA4 binds the linker between RuvC and HNH domains, sterically blocking conformational changes necessary for DNA cleavage (Dong *et al*. 2017; Shin *et al*. 2017; Yang and Patel, 2017). Similar to AcrIIA4, AcrIIA2 prevents target DNA recognition, binding and cleavage. Specifically, AcrIIA2 (Asp71 and Glu72) associates with the PAM-interacting domain (Arg1335 and Arg1333), the WED domain (Lys1107, Glu1108, Ser1109 and Ser1136 of WED domain interact with His37, Asp38, Glu93 and Asp96 of AcrIIA2), the HNH domain (Gln774 and Arg778 interact with Asn19 of AcrIIA2) and the REC2 domain (Lys268 and Asp269 interact with Gln7, Thr28 and Asp30 of AcrIIA2) (Jiang *et al*. 2019; Liu *et al*. 2019). Notably, both AcrIIA4 and AcrIIA2 are not able to bind to SpyCas9 in the absence of a preloaded sgRNA, as sgRNA-binding is required for the formation of the Acr-interaction surface of SpyCas9 (Dong *et al*. 2017; Shin *et al*. 2017; Yang and Patel 2017; Jiang *et al*. 2019; Liu *et al*. 2019).

#### DNA and sgRNA mimicry

Although AcrIIC2 from *Neisseria meningitidis* prophage was previously speculated to associate with catalytic residues of the NmeCas9 HNH domain (Pawluk *et al*. 2016b; Harrington *et al*. 2017), a recent biochemical and structural study revealed interaction with the NmeCas9 bridge helix (BH)-REC1 domain (residues 51–241) (Zhu *et al*. 2019). Indeed, AcrIIC2 forms an homodimer, forming an acidic groove on top of the dimer. The residues in this groove (E18, N23/D24/E25 and residues 109–124) strongly bind to the arginine-rich α helix of BH (residues 56–79; mainly R62, R63, R70 and R73). As such, the AcrIIC2 dimer significantly impedes sgRNA loading to apoNmeCas9, and abrogates dsDNA binding to the sgRNA-NmeCas9-AcrIIC2 complex. Remarkably, AcrIIC2 requires the apo form for effective inhibition, being the first Acr reported to directly interfere with the sgRNA loading to Cas9 (Zhu *et al*. 2019).

#### Dimerization of Cas9

AcrIIC3 from *N. meningitidis* prophage hinders DNA binding and induces NmeCas9 dimerization by associating with the HNH domain and the REC lobe, respectively (Zhu *et al*. 2019). AcrIIC3 (K532-Y540, R557-H563) interacts with a non-conserved region of the NmeCas9 HNH domain opposite to the active site (L58, N60, R33, V34 and D38 among others) (Zhu *et al*. 2019), allowing PAM detection but hampering complete R-loop formation (Harrington *et al*. 2017). Hence, the binding affinity for target DNA is decreased, while DNA cleavage is abrogated. AcrIIC3 additionally associates with the REC lobe, triggering NmeCas9 dimerization with a 2:2 stoichiometry. Each AcrIIC3 molecule binds to the HNH domain as well as the REC lobe of the same or another NmeCas9 molecule, forcing AcrIIC3-Cas9 dimerization and preventing target DNA loading (Zhu *et al*. 2019).

#### Unknown mechanisms

Recently, AcrIIC4 and AcrIIC5 were discovered in a *Haemophilus parainfluenzae* prophage and a *Simonsiella muelleri* transfer element. Both were shown to impede the Cas9:sgRNA complex from binding to the target DNA, following an unknown mode-of-action (Lee *et al*. 2018).

### B) Preventing DNA Cleavage via Interaction with the Cas9 Protein at the Catalytic Site

Like the type I AcrIF3, AcrIIC1 from *N. meningitidis* MGE allows binding of the CRISPR interference complex to the target DNA, though hampering DNA cleavage. Biochemical and structural characterization have revealed that AcrIIC1 specifically binds to the active site of the NmeCas9 HNH domain (D587, H588), blocking cleavage of the target strand and preventing conformational changes necessary for the activation of the RuvC domain, which would theoretically catalyse cleavage of the non-target strand. Thus, the sgRNA-loaded Cas9 remains bound to the target DNA, being trapped in a catalytically inactive state. To achieve high stability of the inter-protein interaction, AcrIIC1 additionally associates with five neighbouring residues of the HNH domain (K549, K551, D598, K603, N616). Similar to AcrIIC1, AcrIIC2 associates with catalytic residues of the NmeCas9 HNH domain, albeit the exact mode-of-action is still elusive (Pawluk *et al*. 2016b; Harrington *et al*. 2017).

### C) Preventing CRISPR-Cas Immunity via Putative Binding to RNA or DNA Molecules

Similar to AcrID1, AcrIIA1 from *L. monocytogenes* prophage J0161a forms a homodimer, though with an unusual two helical-domain structure. The N-terminal domain resembles the HTH motif of transcriptional factors, whilst the CTD adopts an architecture of unknown function. It is anticipated that AcrIIA1 recognizes and associates with heterogeneous RNA molecules to abolish CRISPR-Cas immunity. However, no binding to CRISPR RNA (crRNA), trans-activating RNA (tracrRNA) or their duplex has been observed. AcrIIA1 harbours a positively charged surface around the HTH region, resembling nucleic acid binding motifs of many transcriptional factors that are crucial for RNA and dsDNA recognition. Hence, it would be possible that AcrIIA1 binds to the promoter regions of crRNA or tracrRNA to hinder CRISPR-Cas immunity. However, Cas9 expression levels appeared to be unaffected by AcrIIA1 (Ka *et al*. 2018). The unique structure and function of AcrIIA1 reveals a novel mechanism of action yet unknown among Acr proteins, strengthening our understanding about the versatile and sophisticated ways in which these small proteins may hamper CRISPR-Cas systems (Rauch *et al*. 2017; Ka *et al*. 2018).

### D) Preventing RNA Binding or Cleavage via Interaction with the Cas13b Protein

Although not associated to a phage genome, through the computational pipeline that guided the discovery of subtype VI-B CRISPR-Cas loci, the accessory protein Csx27 was recently found to repress the interference stages of its associated CRISPR-Cas system (Smargon *et al*. 2017). Experimental testing of *Bergeyella zoohelcum* Csx27 (201 aa) has demonstrated an inhibitory effect of the protein when expressed together with different Cas13b proteins, weakening their RNA interference activity. Even though important details on the mechanism of Csx27 remain to be identified, the current findings suggest a broad activity of the protein among type VI-B loci, relating to a possible regulatory mechanism of phage interference (Smargon *et al*. 2017).

## APPLICATIONS OF ANTI-CRISPR PROTEINS

CRISPR-Cas systems have recently been scrutinised for their potential in biotechnological applications. Type II CRISPR-Cas9 systems raise special interest among the scientific community, due to their programmability and specific nuclease activity, representing the most promising tool on genome editing and modulation studied to date (Komor, Badran and Liu 2017). The use of catalytically inactive Cas9 (‘dead’, dCas9) was also previously shown to have powerful biotechnological applications. These include CRISPR interference and activation (CRISPRi and CRISPRa), modification of epigenetic marks and gene expression modulation when fused dCas9 to other metabolic key enzymes (Hilton *et al*. 2015). Other applications encompass dynamic genomic imaging, identification of specific genes or genomic loci, monitoring of gene copy and follow-up of chromatin formation and telomere elongation, when combining site-specific sgRNA molecules with fluorescent tagging of dCas9 (EGFT-dCas9) (Rauch *et al*. 2017; Liu *et al*. 2018). Recently, a Cas9-Assisted Targeting of Chromosome segments (CATCH) was also used for nanopore sequencing of a breast cancer gene (Gabrieli *et al*. 2018). Taken together, the quick emergence of CRISPR-based technologies and the continuous quest for finding new CRISPR-Cas nucleases and variants hereof indicates that more (advanced) applications are expected to be developed in the near future. However, full specificity is crucial in these applications, especially those with therapeutic purposes, as off-target events are still one of the main bottlenecks that limit the efficiency of this technology (Zhang *et al*. 2015).

Deeper insight in Acr inhibitory mechanisms might soon allow for precise temporal, spatial and conditional control of CRISPR-Cas systems through an ‘on-off switch’ regulation. Several AcrIIA and AcrIIC proteins were found to work as ‘off-switches’ of Cas9 activity in human cell lines (Pawluk *et al*. 2016b, Rauch *et al*. 2017). The potential of combining these natural CRISPR inhibitors with Cas9 editing systems, tuning its activity in cellular environments, could result in full optimisation of gene editing processes, substantially decreasing off-target events by allowing Acr proteins to accumulate whenever or wherever editing activity is unwanted (Pawluk *et al*. 2016b; Rauch *et al*. 2017; Shin *et al*. 2017; Hynes *et al*. 2018). Innovation in the use of Acrs to control CRISPR-Cas editing are expected to quickly emerge, as demonstrated by the combination of an AcrIIA4 hybrid with a LOV2 photosensor for the light-mediated control of genome and epigenome editing by CRISPR-Cas9 effectors in human cells (Bubeck *et al*. 2018). Acr-mediated inhibition was also proven to be effective towards the activity of dCas9 processes (Rauch *et al*. 2017; Liu *et al*. 2018; Nakamura *et al*. 2019) as well as the control of genomic circuits and gene editing with Cas9 (Nakamura *et al*. 2019), once again representing a promising tool for optimization of the activity of these CRISPR-based technologies and future therapeutic and biotechnological applications.

Although no research was performed yet on the possible applications of Csx27, this represents the only protein known to date to repress Cas13b, a type VI CRISPR associated protein. In contrast to most other CRISPR-Cas systems currently classified, prokaryotes carrying type VI CRISPR loci are able to target foreign RNA molecules (Smargon *et al*. 2017). The discovery and employment of Acr proteins able to inhibit these systems might allow modulation of future RNA-based biotechnology applications.

Acr proteins might also benefit gene drive technology. Gene drives are powerful tools to eradicate vector-borne diseases, eliminate pests (e.g. agricultural pests and invasive species) and even increase animal welfare. The technology enables the rapid dissemination of genetic mutation(s) through a population by surpassing Mendelian inheritance rules regardless of the fitness-affecting properties of the introduced mutation (Burt 2003). This is accomplished by turning heterozygous organisms into homozygotes through the incorporation of the desired mutated gene flanked by a homing endonuclease (such as CRISPR-Cas) and a corresponding guide RNA (targeting the wild-type gene) in the genome. Upon recognition of the target sequence (i.e. the wild-type gene) on the homologous chromosome, the homing endonuclease will introduce a break at the target site. If this event is followed by homologous recombination, a cassette consisting of the mutated gene flanked by the homing nuclease and the guide RNA will replace the wild-type locus. However, one of the main technological hurdles to overcome is the current inability to effectively control the spreading of a gene drive once out in the environment (DiCarlo *et al*. 2015; Webber, Raghu and Edwards 2015). Acrs can be used as a molecular tool to control this spread. These proteins have been shown to reduce the efficiency of the homing nuclease in a tweakable manner (Basgall *et al*. 2018; Roggenkamp *et al*. 2018). Acrs are also envisioned to enable timed drive activation and to aid anti-gene drives in destroying the original gene drive construct (immunization) (Basgall *et al*. 2018). The latter can be achieved by introducing an *acr* encoding gene instead of a mutated gene, as part of a gene drive. This gene drive should make use of a homing endonuclease which is not suppressed by the respective *acr* gene. Though gene drive efficiency can be partially controlled via sgRNA design (Noble *et al*. 2017; Roggenkamp *et al*. 2018; Yan and Finnigan 2018), incorporation of Acrs in the molecular design to control spreading is advantageous over regulation via sgRNA design since Acrs work directly against the homing endonuclease whereas sgRNA based molecular principles are case specific.

Finally, Acr proteins might represent a powerful tool to enable phage therapy in CRISPR-active hosts. The emergence of multidrug resistant bacteria represents a rising scientific concern due to the possible implications of antibiotic unresponsive infections. Phage therapy poses an interesting alternative to the control of these bacterial infections (Nobrega *et al*. 2015), and Acr-mediated inhibition of active CRISPR-Cas systems might facilitate the employment of known phages that would be otherwise targeted by the bacterial immune system. The possibility of using studied phages, instead of the constant search and characterisation of new ones not yet targeted by the bacterial CRISPR-Cas system, might represent a therapeutic advantage and lead to faster treatment.

## OUTLOOK

A limited number of Acr proteins has been identified so far, but their sequence and structural diversity is already remarkable. The discovery of new Acrs, especially those targeting CRISPR-Cas (sub)types for which Acr proteins have not yet been found, is expected to clarify the number of distinct Acr protein families and how widespread they are. The development of new Acr identification strategies will certainly be required to avoid biases created by current pipelines. Clarification of the mechanisms of multiple Acr proteins, including the characterisation of AcrVA1, AcrVA4 and AcrVA5 (Dong *et al*. 2019; Knott *et al*. 2019) while our study was under review, is expected to fuel the Acr-based fine-tuning of CRISPR-Cas applications, such as gene editing or gene drives.

Because bacteria and phages have co-evolved together for billions of years, it is anticipated that bacteria have developed mechanisms to counteract Acr protein activity. Possible strategies have been hinted, including the accumulation of multiple types of CRISPR-Cas systems in a single cell (e.g. type I-E and I-F systems in *P. aeruginosa*), mutation of *cas* genes (Pausch *et al*. 2017), or silencing of *acr* gene expression. Proper research on the field will certainly increase our understanding on bacterial evolution, and also expand the CRISPR toolbox for biotechnological applications.

## References

[bib1] Barrangou R , Fremaux C, Deveau H et al. CRISPR provides acquired resistance against viruses in prokaryotes. Science. 2007;315:1709–12. 17379808 10.1126/science.1138140

[bib2] Basgall EM , Goetting SC, Goeckel ME et al. Gene drive inhibition by the anti-CRISPR proteins AcrIIA2 and AcrIIA4 in *Saccharomyces cerevisiae*. Microbiology. 2018;164:464–74. 29488867 10.1099/mic.0.000635 PMC5982135

[bib3] Bolotin A , Ouinquis B, Sorokin A et al. Clustered regularly interspaced short palindrome repeats (CRISPRs) have spacers of extrachromosomal origin. Microbiol-Sgm. 2005;151:2551–61. 10.1099/mic.0.28048-0 16079334

[bib5] Bondy-Denomy J , Garcia B, Strum S et al. Multiple mechanisms for CRISPR-Cas inhibition by anti-CRISPR proteins. Nature. 2015;526:136–9. 26416740 10.1038/nature15254 PMC4935067

[bib4] Bondy-Denomy J , Pawluk A, Maxwell KL et al. Bacteriophage genes that inactivate the CRISPR/Cas bacterial immune system. Nature. 2013;493:429–U181. 23242138 10.1038/nature11723 PMC4931913

[bib6] Borges AL , Davidson AR, Bondy-Denomy J The discovery, mechanisms, and evolutionary impact of Anti-CRISPRs. Annual Rev Virol. 2017;4:37–59. 28749735 10.1146/annurev-virology-101416-041616 PMC6039114

[bib7] Borges AL , Zhang JY, Rollins MF et al. Bacteriophage cooperation suppresses CRISPR-Cas3 and Cas9 immunity. Cell. 2018;174:917–25. e910. 30033364 10.1016/j.cell.2018.06.013 PMC6086726

[bib8] Brouns SJJ , Jore MM, Lundgren M et al. Small CRISPR RNAs guide antiviral defense in prokaryotes. Science. 2008;321:960–4. 18703739 10.1126/science.1159689 PMC5898235

[bib9] Brussow H , Canchaya C, Hardt WD Phages and the evolution of bacterial pathogens: from genomic rearrangements to lysogenic conversion. Microbiol Mol Biol Rev. 2004;68:560–602. 15353570 10.1128/MMBR.68.3.560-602.2004 PMC515249

[bib10] Bryson AL , Hwang Y, Sherrill-Mix S et al. Covalent modification of bacteriophage T4 DNA inhibits CRISPR-Cas9. MBio. 2015;6:e00648. 10.1128/mBio.00648-15 PMC4471564 26081634

[bib11] Bubeck F , Hoffmann MD, Harteveld Z et al. Engineered anti-CRISPR proteins for optogenetic control of CRISPR-Cas9. Nat Methods. 2018;15:924–7. 30377362 10.1038/s41592-018-0178-9

[bib12] Burt A . Site-specific selfish genes as tools for the control and genetic engineering of natural populations. P Roy Soc B-Biol Sci. 2003;270:921–8. 10.1098/rspb.2002.2319 PMC1691325 12803906

[bib13] Chopin MC , Chopin A, Bidnenko E Phage abortive infection in lactococci: variations on a theme. Curr Opin Microbiol. 2005;8:473–9. 15979388 10.1016/j.mib.2005.06.006

[bib14] Chowdhury S , Carter J, Rollins MF et al. Structure reveals mechanisms of viral suppressors that intercept a CRISPR RNA-guided surveillance complex. Cell. 2017;169:47–57. e11. 28340349 10.1016/j.cell.2017.03.012 PMC5478280

[bib15] Deltcheva E , Chylinski K, Sharma CM et al. CRISPR RNA maturation by trans-encoded small RNA and host factor RNase III. Nature. 2011;471:602–7. 21455174 10.1038/nature09886 PMC3070239

[bib16] Deveau H , Barrangou R, Garneau JE et al. Phage response to CRISPR-encoded resistance in *Streptococcus thermophilus*. J Bacteriol. 2008;190:1390–400. 18065545 10.1128/JB.01412-07 PMC2238228

[bib17] DiCarlo JE , Chavez A, Dietz SL et al. Safeguarding CRISPR-Cas9 gene drives in yeast. Nat Biotechnol. 2015;33:1250–55. 26571100 10.1038/nbt.3412 PMC4675690

[bib18] Dong Guo M , Wang S, Zhu Y et al. Structural basis of CRISPR-SpyCas9 inhibition by an anti-CRISPR protein. Nature. 2017;546:436–9. 28448066 10.1038/nature22377

[bib19] Dong L , Guan X, Li N et al. An anti-CRISPR protein disables type V Cas12a by acetylation. Nat Struct Mol Biol. 2019;26:308–14. 30936526 10.1038/s41594-019-0206-1

[bib20] Doron S , Melamed S, Ofir G et al. Systematic discovery of antiphage defense systems in the microbial pangenome. Science. 2018;359:6379. 10.1126/science.aar4120 PMC6387622 29371424

[bib21] Drake JW , Charlesworth B, Charlesworth D et al. Rates of spontaneous mutation. Genetics. 1998;148:1667–86. 9560386 10.1093/genetics/148.4.1667 PMC1460098

[bib22] Fagerlund RD , Wilkinson ME, Klykov O et al. Spacer capture and integration by a type I-F Cas1-Cas2-3 CRISPR adaptation complex. Proc Natl Acad Sci. 2017;114:E5122–8. 28611213 10.1073/pnas.1618421114 PMC5495228

[bib23] Forterre P , Prangishvili D. The great billion-year war between ribosome- and capsid-encoding organisms (cells and viruses) as the major source of evolutionary novelties. Ann Ny Acad Sci. 2009;1178:65–77. 19845628 10.1111/j.1749-6632.2009.04993.x

[bib24] Gabrieli T , Sharim H, Fridman D et al. Selective nanopore sequencing of human BRCA1 by Cas9-assisted targeting of chromosome segments (CATCH). Nucleic Acids Res. 2018;46:e87. 29788371 10.1093/nar/gky411 PMC6101500

[bib25] Gandon S Why be temperate: lessons from bacteriophage lambda. Trends Microbiol. 2016;24:356–65. 26946976 10.1016/j.tim.2016.02.008

[bib26] Garneau JE , Dupuis ME, Villion M et al. The CRISPR/Cas bacterial immune system cleaves bacteriophage and plasmid DNA. Nature. 2010;468:67–71. 21048762 10.1038/nature09523

[bib27] Goldfarb T , Sberro H, Weinstock E et al. BREX is a novel phage resistance system widespread in microbial genomes. EMBO J. 2015;34:169–83. 25452498 10.15252/embj.201489455 PMC4337064

[bib28] Guemes AGC , Youle M, Cantu VA et al. Viruses as winners in the game of life. Ann Rev Virol, Vol 3. 2016;3:197–214. 10.1146/annurev-virology-100114-054952 27741409

[bib29] Guo TW , Bartesaghi A, Yang H et al. Cryo-EM structures reveal mechanism and inhibition of DNA targeting by a CRISPR-Cas surveillance complex. Cell. 2017;171:414–26. e412. 28985564 10.1016/j.cell.2017.09.006 PMC5683424

[bib30] Hale C , Kleppe K, Terns RM et al. Prokaryotic silencing (psi) RNAs in *Pyrococcus furiosus*. RNA. 2008;14:2572–9. 18971321 10.1261/rna.1246808 PMC2590957

[bib31] Hale CR , Zhao P, Olson S et al. RNA-guided RNA cleavage by a CRISPR RNA-Cas protein complex. Cell. 2009;139:945–56. 19945378 10.1016/j.cell.2009.07.040 PMC2951265

[bib32] Harrington LB , Doxzen KW, Ma E et al. A broad-spectrum inhibitor of CRISPR-Cas9. Cell. 2017;170:1224–33. e1215. 28844692 10.1016/j.cell.2017.07.037 PMC5875921

[bib33] Haurwitz RE , Jinek M, Wiedenheft B et al. Sequence- and structure-specific RNA processing by a CRISPR endonuclease. Science. 2010;329:1355–8. 20829488 10.1126/science.1192272 PMC3133607

[bib34] He F , Bhoobalan-Chitty Y, Van LB et al. Anti-CRISPR proteins encoded by archaeal lytic viruses inhibit subtype I-D immunity. Nature Microbiol. 2018;3:461–9. 29507349 10.1038/s41564-018-0120-z PMC11249088

[bib35] Hilton IB , D'Ippolito AM, Vockley CM et al. Epigenome editing by a CRISPR-Cas9-based acetyltransferase activates genes from promoters and enhancers. Nat Biotechnol. 2015;33:510–7. 25849900 10.1038/nbt.3199 PMC4430400

[bib36] Hynes AP , Rousseau GM, Agudelo D et al. Widespread anti-CRISPR proteins in virulent bacteriophages inhibit a range of Cas9 proteins. Nature Communications. 2018;9:2919. 10.1038/s41467-018-05092-w PMC6060171 30046034

[bib37] Jackson SA , McKenzie RE, Fagerlund RD et al. CRISPR-Cas: adapting to change. Science. 2017;356:6333. 10.1126/science.aal5056 28385959

[bib38] Jiang F , Liu JJ, Osuna BA et al. Temperature-responsive competitive inhibition of CRISPR-Cas9. Mol Cell. 2019;73:601–10. e605. 30595438 10.1016/j.molcel.2018.11.016 PMC6480404

[bib39] Jiang W , Maniv I, Arain F et al. Dealing with the evolutionary downside of CRISPR immunity: bacteria and beneficial plasmids. PLos Genet. 2013;9:e1003844. 24086164 10.1371/journal.pgen.1003844 PMC3784566

[bib40] Juhala RJ , Ford ME, Duda RL et al. Genomic sequences of bacteriophages HK97 and HK022: pervasive genetic mosaicism in the lambdoid bacteriophages. J Mol Biol. 2000;299:27–51. 10860721 10.1006/jmbi.2000.3729

[bib41] Ka D , Jang DM, Han BW et al. Molecular organization of the type II-A CRISPR adaptation module and its interaction with Cas9 via Csn2. Nucleic Acids Res. 2018;46:9805–15. 30102386 10.1093/nar/gky702 PMC6182153

[bib1_828_1558689730776] Kim I , Jeong M, Ka D et al. Solution structure and dynamics of anti-CRISPR AcrIIA4, the Cas9 inhibitor. Scientific reports. 2018;8:3883. 29497118 10.1038/s41598-018-22177-0 PMC5832863

[bib42] Knott GJ , Thornton BW, Lobba MJ et al. Broad-spectrum enzymatic inhibition of CRISPR-Cas12a. Nat Structural Mol Biol. 2019;26:315–21. 10.1038/s41594-019-0208-z PMC6449189 30936531

[bib43] Komor AC , Badran AH, Liu DR CRISPR-based technologies for the manipulation of eukaryotic genomes. Cell. 2017;169:559. 10.1016/j.cell.2017.04.005 28431253

[bib44] Koonin EV , Dolja VV. A virocentric perspective on the evolution of life. Curr Opin Virol. 2013;3:546–57. 23850169 10.1016/j.coviro.2013.06.008 PMC4326007

[bib45] Koonin EV , Makarova KS, Zhang F. Diversity, classification and evolution of CRISPR-Cas systems. Curr Opin Microbiol. 2017;37:67–78. 28605718 10.1016/j.mib.2017.05.008 PMC5776717

[bib46] Kronheim S , Daniel-Ivad M, Duan Z et al. A chemical defence against phage infection. Nature. 2018;564:283–6. 30518855 10.1038/s41586-018-0767-x

[bib47] Kunne T , Kieper SN, Bannenberg JW et al. Cas3-derived target dna degradation fragments fuel primed CRISPR adaptation. Mol Cell. 2016;63:852–64. 27546790 10.1016/j.molcel.2016.07.011

[bib48] Landsberger M , Gandon S, Meaden S et al. Anti-CRISPR phages cooperate to overcome CRISPR-cas immunity. Cell. 2018;174:908–16. e912. 30033365 10.1016/j.cell.2018.05.058 PMC6086933

[bib49] Lee J , Mir A, Edraki A et al. Potent Cas9 inhibition in bacterial and human cells by AcrIIC4 and AcrIIC5 Anti-CRISPR proteins. MBio. 2018;9:6. 10.1128/mBio.02321-18 PMC6282205 30514786

[bib50] Liu L , Yin ML, Wang M et al. Phage AcrIIA2 DNA mimicry: structural basis of the CRISPR and Anti-CRISPR arms race. Mol Cell. 2019;73:611–20. 30606466 10.1016/j.molcel.2018.11.011

[bib51] Liu XS , Wu H, Krzisch M et al. Rescue of fragile X syndrome neurons by DNA methylation editing of the FMR1 gene. Cell. 2018;172:979–92. e976. 29456084 10.1016/j.cell.2018.01.012 PMC6375087

[bib52] Makarova KS , Wolf YI, Koonin EV. Comparative genomics of defense systems in archaea and bacteria. Nucleic Acids Res. 2013;41:4360–77. 23470997 10.1093/nar/gkt157 PMC3632139

[bib53] Makarova KS , Wolf YI, Snir S et al. Defense islands in bacterial and archaeal genomes and prediction of novel defense systems. J Bacteriol. 2011;193:6039–56. 21908672 10.1128/JB.05535-11 PMC3194920

[bib54] Marino ND , Zhang JY, Borges AL et al. Discovery of widespread type I and type V CRISPR-Cas inhibitors. Science. 2018;362:240–2. 30190308 10.1126/science.aau5174 PMC6520112

[bib55] Marraffini LA , Sontheimer EJ. CRISPR interference limits horizontal gene transfer in staphylococci by targeting DNA. Science. 2008;322:1843–5. 19095942 10.1126/science.1165771 PMC2695655

[bib56] Maxwell KL , Garcia B, Bondy-Denomy J et al. The solution structure of an anti-CRISPR protein. Nature communications. 2016;7:13134. 10.1038/ncomms13134 PMC5062604 27725669

[bib57] Mohanraju P , Makarova KS, Zetsche B et al. Diverse evolutionary roots and mechanistic variations of the CRISPR-Cas systems. Science. 2016;353:6299. 10.1126/science.aad5147 PMC13189112 27493190

[bib58] Mojica FJM , Diez-Villasenor C, Garcia-Martinez J et al. Intervening sequences of regularly spaced prokaryotic repeats derive from foreign genetic elements. J Mol Evol. 2005;60:174–82. 15791728 10.1007/s00239-004-0046-3

[bib59] Nakamura M , Srinivasan P, Chavez M et al. Anti-CRISPR-mediated control of gene editing and synthetic circuits in eukaryotic cells. Nature Commun. 2019;10:194. 30643127 10.1038/s41467-018-08158-x PMC6331597

[bib60] Noble C , Olejarz J, Esvelt KM et al. Evolutionary dynamics of CRISPR gene drives. Sci Adv. 2017;3:e1601964. 28435878 10.1126/sciadv.1601964 PMC5381957

[bib61] Nobrega FL , Costa AR, Kluskens LD et al. Revisiting phage therapy: new applications for old resources. Trends Microbiol. 2015;23:185–91. 25708933 10.1016/j.tim.2015.01.006

[bib62] Pausch P , Muller-Esparza H, Gleditzsch D et al. Structural variation of type I-F CRISPR RNA guided DNA surveillance. Mol Cell. 2017;67:622–32. e624. 28781236 10.1016/j.molcel.2017.06.036

[bib66] Pawluk A , Amrani N, Zhang Y et al. Naturally occurring off-switches for CRISPR-Cas9. Cell. 2016b;167:1829–38. e1829. 27984730 10.1016/j.cell.2016.11.017 PMC5757841

[bib63] Pawluk A , Bondy-Denomy J, Cheung VH et al. A new group of phage anti-CRISPR genes inhibits the type I-E CRISPR-Cas system of *Pseudomonas aeruginosa*. mBio. 2014;5:e00896. 24736222 10.1128/mBio.00896-14 PMC3993853

[bib64] Pawluk A , Shah M, Mejdani M et al. Disabling a type I-E CRISPR-Cas nuclease with a bacteriophage-encoded anti-CRISPR protein. MBio. 2017;8:e01751-17. 10.1128/mBio.01751-17 PMC5727412 29233895

[bib65] Pawluk A , Staals RHJ, Taylor C et al. Inactivation of CRISPR-Cas systems by anti-CRISPR proteins in diverse bacterial species. Nat Microbiol. 2016a;1:16085. 27573108 10.1038/nmicrobiol.2016.85

[bib67] Peng RC , Xu Y, Zhu TF et al. Alternate binding modes of anti-CRISPR viral suppressors AcrF1/2 to Csy surveillance complex revealed by cryo-EM structures. Cell Res. 2017;27:853–64. 28574055 10.1038/cr.2017.79 PMC5518991

[bib68] Pourcel C , Salvignol G, Vergnaud G. CRISPR elements in *Yersinia pestis* acquire new repeats by preferential uptake of bacteriophage DNA, and provide additional tools for evolutionary studies. Microbiol-Sgm. 2005;151:653–63. 10.1099/mic.0.27437-0 15758212

[bib69] Rauch BJ , Silvis MR, Hultquist JF et al. Inhibition of CRISPR-Cas9 with Bacteriophage Proteins. Cell. 2017;168:150–8. 28041849 10.1016/j.cell.2016.12.009 PMC5235966

[bib70] Roggenkamp E , Giersch RM, Schrock MN et al. Tuning CRISPR-Cas9 gene drives in*Saccharomyces cerevisiae*. G3-Genes Genom Genet. 2018;8:999–1018. 10.1534/g3.117.300557 PMC5844318 29348295

[bib71] Rollins MF , Chowdhury S, Carter J et al. Cas1 and the Csy complex are opposing regulators of Cas2/3 nuclease activity. Proc Natl Acad Sci. 2017;114:E5113–21. 28438998 10.1073/pnas.1616395114 PMC5495223

[bib72] Samson JE , Magadan AH, Sabri M et al. Revenge of the phages: defeating bacterial defences. Nat Rev Microbiol. 2013;11:675–87. 23979432 10.1038/nrmicro3096

[bib73] Sano E , Carlson S, Wegley L et al. Movement of viruses between biomes. Appl Environ Microbiol. 2004;70:5842–6. 15466522 10.1128/AEM.70.10.5842-5846.2004 PMC522096

[bib74] Seed KD , Lazinski DW, Calderwood SB et al. A bacteriophage encodes its own CRISPR/Cas adaptive response to evade host innate immunity. Nature. 2013;494:489–91. 23446421 10.1038/nature11927 PMC3587790

[bib75] Semenova E , Jore MM, Datsenko KA et al. Interference by clustered regularly interspaced short palindromic repeat (CRISPR) RNA is governed by a seed sequence. Proc Natl Acad Sci. 2011;108:10098–103. 21646539 10.1073/pnas.1104144108 PMC3121866

[bib76] Shin J , Jiang F, Liu JJ et al. Disabling Cas9 by an anti-CRISPR DNA mimic. Sci Adv. 2017;3:e1701620. 28706995 10.1126/sciadv.1701620 PMC5507636

[bib77] Shmakov SA , Sitnik V, Makarova KS et al. The CRISPR spacer space is dominated by sequences from species-specific mobilomes. MBio. 2017;8:e01397-17. 10.1128/mBio.01397-17 PMC5605939 28928211

[bib78] Smargon AA , Cox DBT, Pyzocha NK et al. Cas13b Is a type VI-B CRISPR-associated RNA-guided RNase differentially regulated by accessory proteins Csx27 and Csx28. Mol Cell. 2017;65:618–30. 28065598 10.1016/j.molcel.2016.12.023 PMC5432119

[bib79] Staals RHJ , Jackson SA, Biswas A et al. Interference-driven spacer acquisition is dominant over naive and primed adaptation in a native CRISPR-Cas system. Nat Commun. 2016;7:12853. 27694798 10.1038/ncomms12853 PMC5059440

[bib80] Stanley SY , Maxwell KL. Phage-encoded anti-CRISPR defenses. Annu Rev Genet. 2018;52:445–64. 30208287 10.1146/annurev-genet-120417-031321

[bib81] Stone NP , Hilbert BJ, Hidalgo D et al. A hyperthermophilic phage decoration protein suggests common evolutionary origin with herpesvirus triplex proteins and an anti-CRISPR protein. Structure. 2018;26:936–47. e933. 29779790 10.1016/j.str.2018.04.008 PMC6277972

[bib82] Sun CL , Barrangou R, Thomas BC et al. Phage mutations in response to CRISPR diversification in a bacterial population. Environ Microbiol. 2013;15:463–70. 23057534 10.1111/j.1462-2920.2012.02879.x

[bib83] Suttle CA . Viruses in the sea. Nature. 2005;437:356–61. 16163346 10.1038/nature04160

[bib84] Tautz D . The discovery of de novo gene evolution. Perspect Biol Med. 2014;57:149–61. 25345708 10.1353/pbm.2014.0006

[bib85] Uribe RV , van der Helm E, Misiakou MA et al. Discovery and characterization of cas9 inhibitors disseminated across seven bacterial phyla. Cell host & microbe. 2019;25:233–41. e235. 30737174 10.1016/j.chom.2019.01.003

[bib86] van Erp PB , Jackson RN, Carter J et al. Mechanism of CRISPR-RNA guided recognition of DNA targets in*Escherichia coli*. Nucleic Acids Res. 2015;43:8381–91. 26243775 10.1093/nar/gkv793 PMC4787809

[bib87] van Houte S , Ekroth AK, Broniewski JM et al. The diversity-generating benefits of a prokaryotic adaptive immune system. Nature. 2016;532:385–8. 27074511 10.1038/nature17436 PMC4935084

[bib88] Vlot M , Houkes J, Lochs SJA et al. Bacteriophage DNA glucosylation impairs target DNA binding by type I and II but not by type V CRISPR-Cas effector complexes. Nucleic Acids Res. 2018;46:873–85. 29253268 10.1093/nar/gkx1264 PMC5778469

[bib89] Vorontsova D , Datsenko KA, Medvedeva S et al. Foreign DNA acquisition by the I-F CRISPR-Cas system requires all components of the interference machinery. Nucleic Acids Res. 2015;43:10848–60. 26586803 10.1093/nar/gkv1261 PMC4678832

[bib90] Wang JY , Ma J, Cheng Z et al. A CRISPR evolutionary arms race: structural insights into viral anti-CRISPR/Cas responses. Cell Res. 2016a;26:1165–8. 27585537 10.1038/cr.2016.103 PMC5113301

[bib91] Wang XF , Yao DQ, Xu JG et al. Structural basis of Cas3 inhibition by the bacteriophage protein AcrF3. Nat Struct Molecular Biol. 2016b;23:868–70. 10.1038/nsmb.3269 27455460

[bib2_82_1558689739780] Watters KE , Fellmann C, Bai HB et al. Systematic discovery of natural CRISPR-Cas12a inhibitors. Science. 2018;362:236–9. 30190307 10.1126/science.aau5138 PMC6185749

[bib92] Webber BL , Raghu S, Edwards OR. Opinion: is CRISPR-based gene drive a biocontrol silver bullet or global conservation threat?. Proc Natl Acad Sci. 2015;112:10565–7. 26272924 10.1073/pnas.1514258112 PMC4553820

[bib94] Westra ER , van Erp PBG, Kunne T et al. CRISPR immunity relies on the consecutive binding and degradation of negatively supercoiled invader dna by cascade and cas3. Mol Cell. 2012;46:595–605. 22521689 10.1016/j.molcel.2012.03.018 PMC3372689

[bib93] Westra ER , van Houte S, Oyesiku-Blakemore S et al. Parasite exposure drives selective evolution of constitutive versus inducible defense. Curr Biol. 2015;25:1043–9. 25772450 10.1016/j.cub.2015.01.065

[bib96] Yang H , Patel DJ. Inhibition mechanism of an anti-CRISPR suppressor AcrIIA4 targeting SpyCas9. Mol Cell. 2017;67:117–27. e115. 28602637 10.1016/j.molcel.2017.05.024 PMC5595222

[bib95] Yan Y , Finnigan GC. Development of a multi-locus CRISPR gene drive system in budding yeast. Sci Rep. 2018;8:17277. 30467400 10.1038/s41598-018-34909-3 PMC6250742

[bib97] Yaung SJ , Esvelt KM, Church GM. CRISPR/Cas9-mediated phage resistance is not impeded by the DNA modifications of phage T4. PLoS One. 2014;9:e98811. 10.1371/journal.pone.0098811 PMC4041780 24886988

[bib98] Zhang XH , Tee LY, Wang XG et al. Off-target effects in CRISPR/Cas9-mediated genome engineering. Mol Ther Nucleic Acids. 2015;4:e264. 26575098 10.1038/mtna.2015.37 PMC4877446

[bib99] Zhu Y , Gao A, Zhan Q et al. Diverse mechanisms of CRISPR-Cas9 inhibition by type IIC anti-CRISPR proteins. Mol Cell. 2019;74:296–309. 30850331 10.1016/j.molcel.2019.01.038 PMC6750902

